# Construction and validation of a presenteeism prediction model for ICU nurses in China

**DOI:** 10.3389/fpubh.2025.1510147

**Published:** 2025-03-03

**Authors:** Jijun Wu, Yuxin Li, Xiaoli Liu, Yuting Fan, Ping Dai, Baixia Chen, Zhenfan Liu, Xian Rong, Xiaoli Zhong

**Affiliations:** ^1^Department of Nursing, Deyang People’s Hospital, Deyang, China; ^2^School of Nursing, North Sichuan Medical College, Nanchong, China; ^3^Department of Cardiology, Deyang People’s Hospital, Deyang, China; ^4^Sichuan Nursing Vocational College, Chengdu, China

**Keywords:** ICU, nurses, presenteeism, risk factors, column-line diagram, risk prediction model

## Abstract

**Background:**

Presenteeism, also known as impaired health productivity, refers to the condition of impaired productivity of an individual due to physiological or mental health problems. ICU, as a place of intensive care for patients with acute and critical illnesses, nurses have long faced the nature of work with high loads, high pressures, and high intensities, which makes them a high prevalence group of presenteeism. Presenteeism not only affects the physical and mental health and work wellbeing of nurses but also reduces the quality of nursing services and affects the life safety of patients, such as increasing the risk of falls during hospitalization, increasing the risk of medication errors, and prolonging the hospitalization time of patients. Therefore, early identification and targeted interventions are crucial to reduce presenteeism among ICU nurses.

**Objective:**

This study aimed to construct and validate a predictive model for presenteeism among ICU nurses.

**Design:**

A cross-sectional study.

**Methods:**

1,225 ICU nurses were convened from January to April 2023 from 25 tertiary and secondary hospitals in Sichuan Province, China. ICU nurses were randomly divided into a development set (*n* = 859) and a validation set (*n* = 366) according to a 7:3 ratio. Univariate and multifactorial logistic regression analyses were used to determine the influencing factors for presenteeism, and R software was used to construct a column-line graph prediction model. The differentiation and calibration of the predictive model were evaluated by the area under the curve of subjects’ work characteristics (ROC) and the Hosmer-Leme-show test, and the clinical decision curve evaluated the clinical validity of the predictive model.

**Results:**

The presenteeism rate of ICU nurses in the development set was 76.8%. Multifactorial logistic regression analysis showed that independent factors affecting ICU nurses’ presenteeism included income per month, physical health status, job satisfaction, perceived work stress, perceived social support, transformational leadership, and occupational coping self-efficacy. In the development set and validation set, the area under the ROC curve was 0.821 and 0.786, respectively; the sensitivity and specificity were 80.6, 69.8 and 80.9%, 65.1%, respectively; the Hosmer-Lemeshow goodness-of-fit was *χ*^2^ = 8.076 (*p* = 0.426) and *χ*^2^ = 5.134 (*p* = 0.743), respectively, and the model had relatively good discrimination and consistency. The clinical decision curve showed that the model had good clinical validity.

**Conclusion:**

The predictive model of presenteeism risk for ICU nurses constructed in this study has good predictive ability. The model can effectively identify ICU nurses with high presenteeism and provide a reference basis for developing targeted interventions to reduce presenteeism among ICU nurses.

## 1 Introduction

As a place for centralized treatment and care of patients with various acute and critical illnesses, the intensive care unit (ICU) requires ICU nurses to provide continuous care for patients in a relatively closed work environment. ICU is also considered a workplace with long hours, high work intensity, heavy medical responsibilities, reversal of work days and nights, and a relatively closed work environment (1). Nurses are on the frontline of observing patients’ conditions and implementing treatments for an extended period. They need to be highly concentrated and have keen observation ability and quick decision-making ability, which negatively impacts nurses’ physical and mental health, such as empathy fatigue, burnout, and sub-health symptoms (2–4). In addition, ICUs are often confronted with patient resuscitation and death, and death stimuli are considered one of the traumatic events for nurses, with which empathizing with nurses may trigger complex emotional changes and negatively affect their physiology, psychology, and society (5). A meta-analysis of a global sample of 14 countries showed that the detection rate of presenteeism among nurses was about 49.2% (6). Our previous study also showed that the incidence of presenteeism among ICU nurses in China was about 55.4% (7). Therefore, identifying and solving the problem of presenteeism among ICU nurses is an important research area in nursing human resource management.

Presenteeism exists in many professions, but it is particularly prominent in the healthcare field (8). Prof. Copper first defined it in 1996 as “a situation in which an individual continues to work despite a decline in productivity due to illness or prolonged working hours” (9). Presenteeism is considered an occupational hazard, which is caused by physical and mental health problems that prevent nurses from fully engaging in their work, resulting in a decrease in work efficiency, which is mainly manifested in lack of concentration, lack of nurse–patient communication, and decreased awareness of occupational safety, which seriously affects the quality of care, patient safety and patient experience (10–12). In addition, some studies have also shown that presenteeism has a particular impact on nurses’ work happiness, work performance and willingness to leave (13–15). At the same time, presenteeism can also cause substantial economic losses. Studies have shown that the United States suffers an annual damage of about 36 billion dollars due to presenteeism (63), and China loses about 4.38 billion dollars annually (16). Therefore, it is essential to identify the risk of presenteeism among ICU nurses early and take targeted interventions to reduce presenteeism among nurses.

To date, screening for presenteeism among nurses has relied on a variety of assessment tools to identify populations with high levels of presenteeism, such as the Stanford Presenteeism Scale-6 (17) and the Work Limitations Questionnaire (18). The Stanford Presenteeism Scale-6 (SPS-6) is a tool designed to evaluate an individual’s capacity to maintain productivity at work despite health challenges. It can serve as a marker for presenteeism by assessing an individual’s ability to concentrate and complete work tasks effectively. The Work Limitations Questionnaire (WLQ) is another instrument designed to assess limitations in work performance due to health problems, including limitations in ability to manage time, physical demands, and psychological or social demands. While these two instruments can broadly assess an individual’s presenteeism, most of these scales are generalizable, highly subjective, and have limited utility in identifying those at high risk for presenteeism. Therefore, developing a set of predictive models for ICU nurses with good predictive ability is of great clinical importance. However, current studies on presenteeism among Chinese ICU nurses are limited to cross-sectional surveys, and tools have yet to be retrieved on presenteeism risk prediction. Previous studies have shown that some common factors such as nurses’ job stress, nurse leaders’ leadership, transformational leadership, uncivilized behavior in the workplace, health status, perceived social support, and occupational coping self-efficacy are considered to be closely related to presenteeism (19–22). Among them, transformational leadership refers to the promotion of innovation and personal growth by motivating and inspiring subordinates, thereby increasing employees’ job satisfaction and organizational commitment. Study has shown that transformational leadership helps to increase nurses’ job satisfaction and work engagement, which in turn reduces the likelihood of presenteeism (23). In addition, high levels of perceived social support, an important external resource, can significantly reduce work stress, increase psychological resources, and enable nurses to cope more effectively with work challenges (24). When nurses feel supported, they are more likely to maintain high levels of performance and reduce presenteeism due to emotional exhaustion or burnout. While occupational coping self-efficacy serves as an intrinsically positive quality, nurses with higher occupational coping self-efficacy are more likely to adopt positive strategies to meet work challenges, demonstrate greater adaptability and resilience, contribute to higher levels of productivity, and reduce presenteeism due to feelings of powerlessness or frustration (21). In addition, some job-related characteristics such as title, position, years of experience, human resource allocation, shift work, and financial income were also significantly associated with presenteeism (25). Besides, some demographic information such as age, gender, marital status, and the highest level of education were also correlated with presenteeism (26). These influences should be considered when identifying people who may have presenteeism.

Presenteeism is a decrease in productivity and quality of work due to impaired physical and mental health; therefore, presenteeism can be recognized and prevented early. Early assessment and targeted intervention for ICU nurses are crucial in reducing presenteeism. However, the research on presenteeism in nursing human resource management in China is still in its infancy. It is mainly limited to partial investigation of the status quo and analysis of influencing factors, with insufficient knowledge of presenteeism among nurses, resulting in limited ability to assess and effectively intervene in the assessment of presenteeism among nurses. Given the possible influences on presenteeism among clinical nurses, there is a need to improve the identification of those at high risk of presenteeism. Risk prediction models are based on multiple risk factors and are used to identify high-risk populations through mathematical modeling and optimization, thus effectively supporting early targeted intervention. Predictive models have been successfully applied to other aspects of human resource management, such as risk prediction models for nurses with sleep disorders, compassionate fatigue, etc. (27, 28). In addition, column-line plots, which predict the probability of occurrence of outcome events through quantitative scoring of independent influences, have improved prediction accuracy, are effective in personalizing predictions, and have been shown to outperform traditional prediction models significantly (29). However, to our knowledge, no risk prediction model has been developed to predict the occurrence of presenteeism among ICU nurses.

Social Cognitive Theory (SCT) is a theoretical framework proposed by Bandura that emphasizes the interactions between individual behavior, environmental factors, and cognitive factors (30). The theory suggests that an individual’s behavioral performance is influenced not only by internal cognitions and beliefs, but also by the external environment and the behavior of others. The theory is widely used in the fields of education, health behavior, career development, etc., which can deeply explore individual behavior and social phenomena, and provide scientific guidance for practice. Therefore, based on social cognitive theory, this study explored the relationship with presenteeism in terms of internal factors (e.g., occupational coping self-efficacy) and external factors (e.g., transformational leadership, and perceived social support), and constructed a risk prediction model suitable for the presenteeism of ICU nurses in China, in order to provide an opportunity for early identification and timely intervention with the high-risk group, thereby reducing the presenteeism of ICU nurses, improving the work efficiency and quality of nursing services, and ensuring the quality of care provided by ICU nurses. This will provide a reference basis for early identification and timely intervention with the high-risk group, thereby reducing presenteeism of ICU nurses, improving their work efficiency and quality of nursing services, and ensuring patient safety.

This study was organized around the following three hypotheses:

Hypothesis 1: Presenteeism among ICU nurses differs in sociodemographic characteristics.

Hypothesis 2: Presenteeism among ICU nurses is related to transformational leadership, occupational coping self-efficacy, and perceived social support.

Hypothesis 3: A predictive model of presenteeism among ICU nurses will identify at-risk ICU nurses who have not yet developed symptoms of presenteeism.

## 2 Methods

### 2.1 Study design and participants

This study was a cross-sectional study. A questionnaire survey of ICU nurses who met the inclusion criteria in 25 secondary and tertiary hospitals in Sichuan Province, China, was conducted from January to April 2023 using a convenience sampling method. The 25 hospitals surveyed were from five regions of Sichuan Province (North Sichuan, South Sichuan, East Sichuan, South Sichuan, and Chengdu), with four tertiary hospitals and one secondary hospital randomly selected from each region. All investigators signed an electronic informed consent form. Inclusion criteria: obtaining the professional qualification certificate for nurses and working in ICU clinics for more than 1 year; no history of alcohol or drug addiction or mental disorder; no history of medications related to mental diseases; informed consent and voluntary participation in this study. Exclusion criteria: those who are currently absent from work on sick leave, maternity leave, or personal leave; internship, regulation training, and conducting nurses.

The number of events (EPV) for each variable in the logistic regression analysis was used to estimate the sample size for this study (31). The model predictions for this study were estimated to consist of 6–7 predictors. According to the 10 EPV principle, one of our previous studies showed that the incidence of presenteeism among Chinese ICU nurses was about 55.4% (7). Considering 20% invalid questionnaires, the minimum sample size should be 10 × 6 ÷ 55.4% × (1 + 20%) ≈ 130, and the maximum sample size should be 10 × 7 ÷ 55.4% × (1 + 20%) ≈ 152. 1,225 valid questionnaires were finally returned in this study, which met the above requirements.

### 2.2 Data collection

Questionnaire Star was used to create and distribute electronic questionnaires for this study. With the support of the director of the nursing department of each hospital, an ICU nurse manager was identified as the survey liaison for this study. Before the survey, all the survey liaisons identified in this study were given uniform training to explain the purpose, significance, method of completion, and precautions for completion of this study. Using the ICU nurses’ monthly centralized theoretical learning time, the electronic questionnaire link was sent to the ICU nurses’ WeChat group for centralized completion by the survey liaison at a unified point in time, and the investigator conducted on-site supervision and answered any questionable entries. To ensure the completeness of the questionnaire, all options were set as mandatory questions. To avoid duplication, each IP address could only be entered once. At the end of the survey, the collected questionnaires were evaluated, and questionnaires with apparent regularity of completion and logical errors were excluded.

### 2.3 Measures

#### 2.3.1 Socio-demographic characteristics

This study included 20 socio-demographic variables, mainly gender, age, marital and childbearing status, highest level of education, title, and position.

#### 2.3.2 Stanford presenteeism scale

The Stanford Presenteeism Scale, as developed by Koopman et al., was utilized in this study. The scale comprises two dimensions: completion of work and avoidance of distractions, with a total of six items. A five-point Likert scale was employed, with scores ranging from 1 to 5, from “strongly disagree” to “strongly agree,” with entries 5 and 6 reversed, and a total score ranging from 6 to 30, with higher scores indicating a more serious manifestation of presenteeism. The median score of 16 on the scale was employed to categorize low and high levels of presenteeism. The Cronbach’s alpha coefficient for the scale was 0.860. In this study, the Cronbach’s alpha coefficient for the scale was 0.896 (17).

#### 2.3.3 Transformational leadership scale

The Transformational Leadership Scale, as developed by Li et al., was utilized in this study. The scale comprises 26 items distributed across four dimensions: vision and motivation, moral example, leadership charisma, and personalized care. A five-point Likert scale was employed, with scores ranging from one to five, from “strongly disagree” to “strongly agree,” and a total score of 26–130. Higher scores indicated a higher degree of perceived transformational leadership behavior. The Cronbach’s alpha coefficient for the scale is 0.928, while the Cronbach’s alpha coefficient for the scale utilized in this study is 0.906 (32).

#### 2.3.4 Occupational coping self-efficacy scale

The Occupational Coping Self-Efficacy Scale, as developed by Pisanti et al., was utilized in this study. The scale comprises nine items, which can be grouped into two dimensions: individual occupational burden and relationship difficulties. The scale was rated on a 5-point Likert scale, with scores ranging from 1 to 5, from “very inconsistent” to “very consistent,” and the total score ranging from 9 to 45, with higher scores indicating higher self-efficacy for occupational coping. The Cronbach’s alpha coefficient of the scale was 0.882, and the Cronbach’s alpha coefficient of this scale was 0.899 in this study (33).

#### 2.3.5 Perceived social support scale

The Perceived Social Support Scale, as developed by Blumenthal et al., was utilized in this study. The scale comprises three dimensions: family support, friend support, and other support, with a total of 12 items. A seven-point Likert scale was employed, with scores ranging from one to seven, from “strongly disagree” to “strongly agree,” and a total score of 12–84. A higher score indicates a greater level of perceived social support. The Cronbach’s alpha coefficient for the scale is 0.912, and the Cronbach’s alpha coefficient for the scale in this study is 0.910 (34).

### 2.4 Statistical analysis

SPSS 25.0 and R 4.4.0 were used to analyze the data statistically. Measurement information conforming to normal distribution was described by mean ± standard deviation, and comparisons between groups were made by t-test and analysis of variance; non-normally distributed measurements were expressed as median and quartiles and statistically analyzed by the Mann–Whitney U test; and counting information was expressed as frequencies and percentages, and statistically analyzed by the *χ*^2^ test or Fisher’s exact probability method for statistical analysis. The multivariate logistic regression analysis included Independent variables that were statistically significant (*p* < 0.05) in the univariate analysis. The bootstrap method was used to validate the model internally. The area under the subject’s work characteristics (ROC) curve was used to assess the discrimination of the model, the calibration curve to evaluate the calibration of the model, and the clinical decision curve to determine the clinical validity and *α* = 0.05 was used as the level of the test.

### 2.5 Ethical considerations

The study followed the Declaration of Helsinki and was approved by the Ethics Committee of Deyang People’s Hospital (2021-04-056-K01).

## 3 Results

### 3.1 Characteristics of participants

In this study, 1,225 ICU nurses were randomly divided into a development set (*n* = 859) and a validation set (*n* = 366) according to a 7:3 ratio. Of the 859 ICU nurses in the development set, 77 (9.0%) were male, and 782 (91.0%) were female; 354 (41.2%) were aged ≤29 years, 455 (53.0%) were aged 30–39 years, 45 (5.2%) were aged 40–49 years, and 5 (0.6%) were aged ≥50 years; and of the 366 ICU nurses in the validation group, 38 (10.4%) were male and 328 (89.6%) were female, 328 (89.6%) were female; 148 (40.4%) were ≤29 years old, 195 (53.3%) were 30–39 years old, 21 (5.7%) were 40–49 years old, and 2 (0.5%) were ≥50 years old. The rest of the general information is shown in Table 1.

**Table 1:** Characteristics of the participants.

Variable	Development set (*n* = 859)		Validation set (*n* = 366)	
	*n*	%	*n*	%
Gender				
Male	77	9.0	38	10.4
Female	782	91.0	328	89.6
Age (year)				
≤29	354	41.2	148	40.4
30–39	455	53.0	195	53.3
40–49	45	5.2	21	5.7
≥50	5	0.6	2	0.5
Marital or childbearing status				
Unmarried	241	28.1	118	32.2
Married with no children	110	12.8	39	10.7
Married with children	491	57.2	201	54.9
Divorced/widowed	17	2.0	8	2.2
Hospital level				
Secondary hospitals	37	4.3	18	4.9
Tertiary hospitals	822	95.7	348	95.1
Highest education				
Junior college and below	140	16.3	49	13.4
Undergraduate	709	82.5	308	84.2
Master degree or above	10	1.2	9	2.5
Professional title				
Nurse	150	17.5	63	17.2
Nurse practitioner	418	48.7	181	49.5
Charge nurse	276	32.1	114	31.1
Associate nurse practitioner and above	15	1.7	8	2.2
Position				
Clinical nurse	719	83.7	299	81.7
Nursing team leader	101	11.8	43	11.7
Head nurse	39	4.5	24	6.6
Years of ICU work (year)				
<6	354	41.2	143	39.1
6–10	288	33.5	106	29.0
>10	217	25.3	117	32.0
Income per month (RMB)				
<6,000	167	19.4	92	25.1
6,001–8,000	366	42.6	135	36.9
8,001–11,000	238	27.7	93	25.4
>11,001	88	10.2	46	12.6
Type of contract				
Professional preparation	139	16.2	70	19.1
Labor contract	720	83.8	296	80.9
Physical health condition				
Good	383	44.6	194	53.0
General	409	47.6	150	41.0
Worse	67	7.8	22	6.0
Suffering from chronic disease				
Yes	136	15.8	56	15.3
No	723	84.2	310	84.7
Suffering from chronic pain				
Yes	258	30.0	95	26.0
No	601	70.0	271	74.0
Fatigue				
Yes	308	35.9	118	32.2
No	551	64.1	248	67.8
Daily sleeping hours (h)				
≤6	218	25.4	95	26.0
>6	641	74.6	271	74.0
Job satisfaction				
Satisfaction	598	69.6	241	65.8
General	203	23.6	90	24.6
Dissatisfied	58	6.8	35	9.6
Perceived work stress				
Low	14	1.6	7	1.9
Moderate	298	34.7	170	46.4
High	547	63.7	189	51.6
ICU human resource allocation				
<1:2.5–3	488	56.8	197	15.0
=1:2.5–3	224	26.1	114	31.1
>1:2.5–3	147	17.1	55	15.0
Night shift				
Yes	779	90.7	327	89.3
No	80	9.3	39	10.7
Experienced workplace violence				
Yes	340	39.6	129	35.2
No	519	60.4	237	64.8

### 3.2 Current status of the incidence of presenteeism among ICU nurses

In the development set, there were 660 high presenteeism with a median cutoff of a total presenteeism score of 16 (17), and the ICU nurses had a presenteeism rate of 76.8%.

### 3.3 Univariate analysis of presenteeism among ICU nurses

Among the ICU nurses in the development set, the results of the univariate analysis showed that the level of the hospital where they worked, years of ICU work, income per month, physical health condition, daily sleeping hours, job satisfaction, perceived job stress, whether they worked shifts, whether they suffered from violence in the workplace, perceived social support, transformational leadership, and occupational coping self-efficacy were the factors influencing presenteeism among the ICU nurses (*p* < 0.05). See Table 2.

**Table 2:** Univariate analysis of presenteeism among ICU nurses.

Variable	Presenteeism		Statistics	*p* value
	No (*n* = 199)	Yes (*n* = 660)		
Gender				
Male	22 (11.1)	55 (8.3)	1.388	0.239
Female	177 (88.9)	605 (91.7)		
Age (year)				
≤29	83 (41.7)	271 (41.1)	4.431	0.219
30–39	99 (49.7)	356 (53.9)		
40–49	16 (8.0)	29 (4.4)		
≥50	1 (0.5)	4 (0.6)		
Marital or childbearing status				
Unmarried	52 (26.1)	189 (28.6)	6.259	0.100
Married with no children	25 (12.6)	85 (12.9)		
Married with children	122 (61.3)	369 (55.9)		
Divorced/widowed	0	17 (2.6)		
Hospital level				
Secondary hospitals	14 (7.0)	23 (3.5)	4.676	0.031
Tertiary hospitals	185 (93.0)	637 (96.5)		
Highest education				
Junior college and below	40 (20.1)	100 (15.2)	3.581	0.167
Undergraduate	158 (79.4)	551 (3.5)		
Master degree or above	1 (0.5)	9 (1.4)		
Professional title				
Nurse	32 (16.1)	118 (17.9)	6.385	0.094
Nurse practitioner	110 (55.3)	308 (46.7)		
Charge nurse	52 (26.1)	224 (33.9)		
Associate nurse practitioner and above	5 (2.5)	10 (1.5)		
Position				
Clinical nurse	167 (83.9)	552 (83.6)	1.910	0.385
Nursing team leader	20 (10.1)	81 (12.3)		
Head nurse	12 (6.0)	27 (4.1)		
Years of ICU work (year)				
<6	86 (43.2)	268 (40.6)	8.635	0.013
6–10	51 (25.6)	237 (35.9)		
>10	62 (31.2)	155 (23.5)		
Income per month (RMB)				
<6,000	36 (18.1)	131 (19.8)	12.012	0.007
6,001–8,000	83 (41.7)	283 (42.9)		
8,001–11,000	47 (23.6)	191 (28.9)		
>11,001	33 (16.6)	55 (8.3)		
Type of contract				
Professional preparation	37 (18.6)	102 (15.5)	1.110	0.292
Labor contract	162 (81.4)	558 (84.5)		
Physical health condition				
Good	142 (71.4)	241 (36.5)	78.665	<0.001
General	55 (27.6)	354 (53.6)		
Worse	2 (1.0)	65 (9.8)		
Suffering from chronic disease				
Yes	25 (12.6)	111 (16.8)	2.078	0.149
No	174 (87.4)	549 (83.2)		
Suffering from chronic pain				
Yes	55 (27.6)	203 (30.8)	0.708	0.400
No	144 (72.4)	457 (69.2)		
Fatigue				
Yes	68 (34.2)	240 (36.4)	0.320	0.572
No	131 (65.8)	420 (63.6)		
Daily sleeping hours (h)				
≤6	39 (19.6)	179 (27.1)	4.570	0.033
>6	160 (80.4)	481 (72.9)		
Job satisfaction				
Satisfaction	122 (61.3)	476 (72.1)	8.456	0.015
General	60 (30.2)	143 (21.7)		
Dissatisfied	17 (8.5)	41 (6.2)		
Perceived work stress				
Low	12 (6.0)	2 (0.3)	116.369	<0.001
Moderate	120 (60.3)	178 (27.0)		
High	67 (33.7)	480 (72.7)		
ICU human resource allocation				
<1:2.5–3	107 (53.8)	381 (57.7)	5.499	0.064
=1:2.5–3	64 (32.2)	160 (24.2)		
>1:2.5–3	28 (14.1)	119 (18.0)		
Night shift				
Yes	172 (86.4)	607 (92.0)	5.551	0.018
No	27 (13.6)	53 (8.0)		
Experienced workplace violence				
Yes	55 (27.6)	285 (43.2)	15.447	<0.001
No	144 (72.4)	375 (56.8)		
Perceived social support	66.44 ± 9.14	59.39 ± 12.20	2.585	<0.001
Transformational leadership	110.08 ± 16.31	99.19 ± 21.11	3.734	<0.001
Occupational coping self-efficacy	33.20 ± 6.79	28.65 ± 6.88	7.850	<0.001

### 3.4 Logistic regression analysis of presenteeism of ICU nurses

Logistic regression analysis was performed by taking the statistically significant factors in the univariate analysis as the independent variables. Whether there was substantial presenteeism among ICU nurses as the dependent variable, and the results showed that average monthly personal income exceeding 11,001 yuan (RMB), along with general job satisfaction, along with higher scores perceived social support, transformational leadership, and occupational coping self-efficacy, served as protective factors for presenteeism among ICU nurses. Conversely, general or worse physical health and moderate to high perceived work stress emerged as risk factors for presenteeism among ICU nurses (*p* < 0.05). See Table 3.

**Table 3:** Logistic regression analysis of presenteeism of ICU nurses.

Predictors	*β*	SE	Wald	OR (95%CI)	*p*
(Constant)	3.745	1.294	8.373	–	0.004
Income per month					0.040
<6,000				1 (ref)	
6,001–8,000	−0.497	0.289	3.020	0.608 (0.347–1.066)	0.082
8,001–11,000	−0.067	0.309	0.047	0.935 (0.510–1.715)	0.829
>11,001	−0.809	0.360	5.040	0.445 (0.220–0.902)	0.025
Physical health condition					<0.001
Good				1 (ref)	
General	0.868	0.212	16.753	2.382 (1.572–3.610)	<0.001
Worse	1.542	0.755	4.169	4.675 (1.064–20.542)	0.041
Job satisfaction					0.017
Satisfaction				1 (ref)	
General	−0.566	0.218	6.727	0.568 (0.370–0.871)	0.009
Dissatisfied	−0.593	0.362	2.682	0.553 (0.272–1.124)	0.101
Perceived work stress					<0.001
Low				1 (ref)	
Moderate	1.735	0.845	4.215	5.667 (1.082–29.682)	0.040
High	3.115	0.848	13.493	22.532 (4.276–118.746)	<0.001
Perceived social support	−0.026	0.011	5.862	0.975 (0.954–0.995)	0.015
Transformational leadership	−0.022	0.006	12.205	0.979 (0.967–0.991)	<0.001
Occupational coping self-efficacy	−0.031	0.015	4.253	0.969 (0.941–0.998)	0.039

### 3.5 Construction of ICU nurses’ presenteeism prediction model

**Figure 1:**
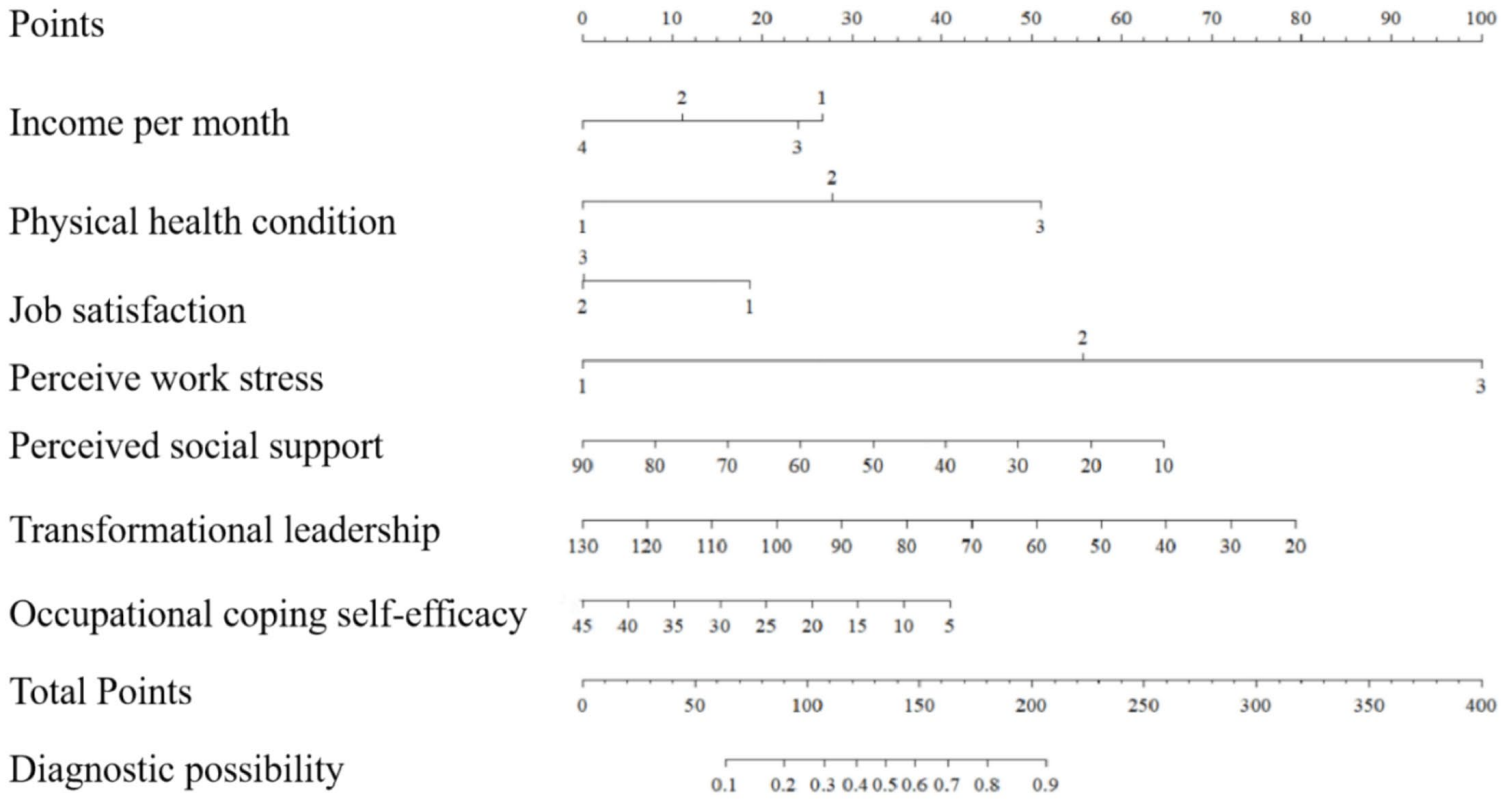
Column line diagram of the predictive model for the risk of presenteeism among ICU nurses.

Based on the results of multifactor logistic regression analysis, a column-line graph of ICU nurses’ presenteeism was constructed (see Figure 1). The column-line graph was used to identify the score corresponding to each factor in the graph. The scores were summed up as the total score, and the probability of occurrence of ICU nurses’ presenteeism was obtained based on the total score on the risk axis.

For example, an ICU nurse in a hospital may have an average personal monthly income of 8,001–11,000 yuan (RMB), poor health, average job satisfaction, high perceived job stress, 50 points for perceived social support, 90 points for transformational leadership, and 25 points for occupational coping self-efficacy. In the column-line diagram model, each variable is assigned a specific score on the rating scale. Therefore, the average monthly personal income (8,001–11,000) is equivalent to 26 points, physical health (average) is equivalent to 28 points, job satisfaction (dissatisfaction) is equivalent to 20 points, and perceived job stress (average) is equivalent to 56 points. The variables are as follows: average monthly personal income (8,001–11,000) = 26 points, physical health (average) = 28 points, job satisfaction (dissatisfaction) = 20 points, perceived job stress (average) = 56 points, perceived social support (60 points) = 24 points, transformational leadership (90 points) = 30 points, and occupational coping self-efficacy (25 points) = 20 points. The total score was 204. The predicted probability of presenteeism was obtained by summing the scores for each variable and drawing a vertical line across the total score. Therefore, the ICU nurse exhibited a 90% probability of developing a nomogram model of presenteeism.

### 3.6 Evaluation and validation of a predictive model of presenteeism for ICU nurses

**Figure 2:**
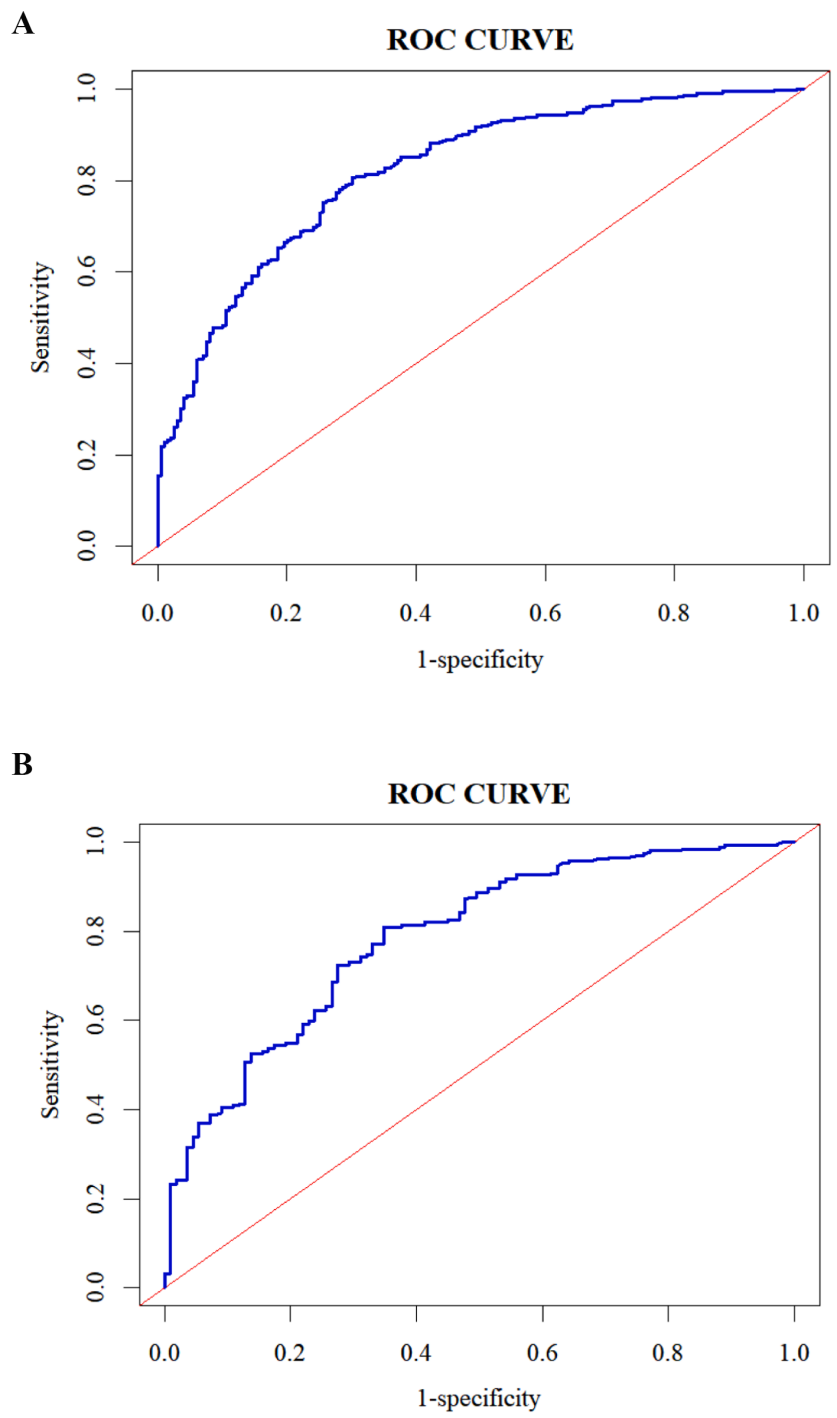
ROC curve of the predictive model for the risk of presenteeism of ICU nurses in both groups. **(A)** Development set; **(B)** validation set.

**Figure 3:**
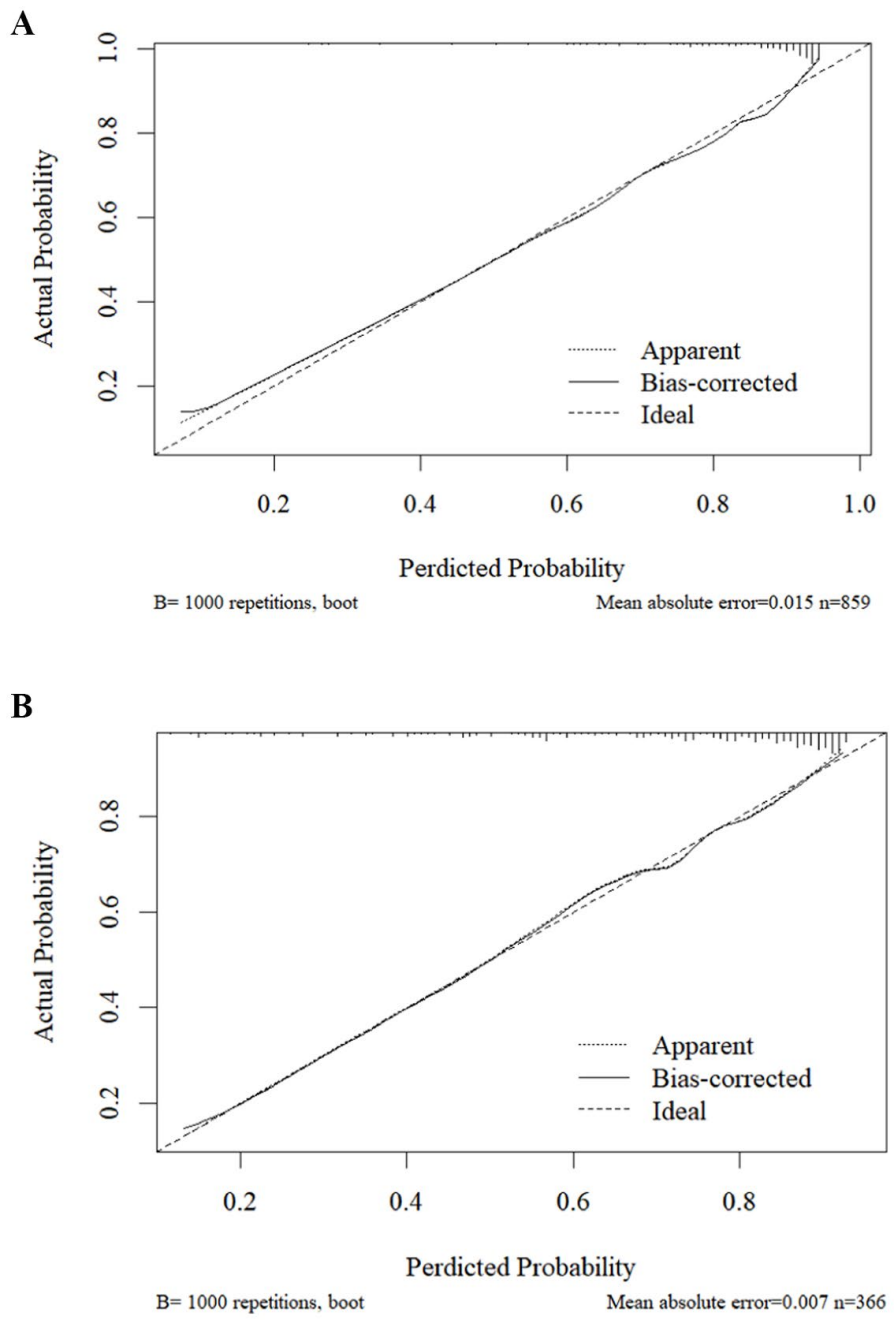
Calibration curve of the predictive model for the risk of presenteeism for ICU nurses in both groups. **(A)** Development set; **(B)** validation set.

The ROC curve was plotted according to the relationship between the predicted probability of the model and the level of presenteeism of ICU nurses, and the area under the curve was 0.821 (95% CI 0.788–0.853, *p* < 0.001), with the optimal critical value of 0.709, and the sensitivity and specificity of the predictive model were 80.6 and 69.8%, respectively, which indicated that the model had an excellent discriminatory degree, see Figure 2A. The calibration curve was drawn using the Bootstrap method for internal validation of the column plot predictive model. Bootstrap method to repeat the sampling 1,000 times, draw the calibration curve to validate the prediction model of the column line graph internally, and the calibration curve is close to the ideal curve, see Figure 3A; the result of the Hosmer-Le-meshow goodness-of-fit test is *χ*^2^ = 8.076 (*p* = 0.426), which indicates that the prediction model has a better fitting effect, good calibration, and that the predicted probability and the actual risk have good consistency.

In the validation set, the area under the ROC curve of the model was 0.786 (95% CI 0.736–0.786, *p* < 0.001), with an optimal critical value of 0.642, and the sensitivity and specificity of the predictive model were 80.9 and 65.1%, respectively, which indicated that the model had an excellent discriminatory degree, see Figure 2B. The result of the Hosmer-Le-meshow goodness-of-fit test was *χ*^2^ = 5.134 (*p* = 0.743). The calibration curve agreed with the ideal curve, see Figure 3B.

**Figure 4:**
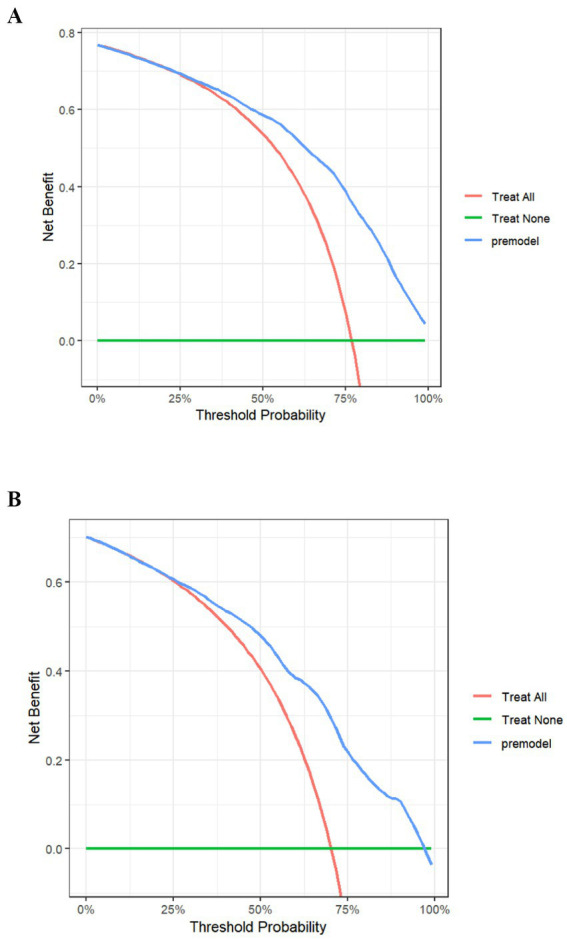
Clinical decision curve of the presenteeism risk prediction model for ICU nurses in both groups. **(A)** Development set; **(B)** validation set.

The clinical decision curve evaluates the clinical validity of the predictive model. The horizontal coordinate of the clinical decision curve is the threshold probability and the vertical coordinate is the standardized gain. At a given threshold likelihood, the predictive model’s decision curve is above the reference line, which may indicate that the model’s clinical utility is generally better. In this study, when the model predicts that all nurses do not experience presenteeism and no measures are taken, the net benefit ratio is 0, which is indicated by the green solid line; when the model predicts that all nurses experience presenteeism and measures are taken, the slope of the net benefit ratio is negative, which is indicated by the red solid line; when the decision curve of the prediction model is further away from the green solid line and the red solid line and nearer to the upper-right corner, it indicates that the model’s clinical validity is better. The blue curves of the model’s predicted benefit scenarios in this study are almost entirely above the other curves, indicating that the model’s decisions can benefit nurses. See Figure 4.

## 4 Discussion

### 4.1 Current status of presenteeism among ICU nurses

Presenteeism is prevalent in the nursing population, is 3–4 times more prevalent than in other professions, and has varying degrees of negative impact on healthcare organizations, nurses, and patients (35). In this study, the incidence of presenteeism among ICU nurses was 76.8%, which was higher than the overall incidence of presenteeism among nurses globally (49.2%) (6). This finding indicates that the incidence of presenteeism among ICU nurses in China was relatively high. Potential reasons for this phenomenon can be analyzed as follows: First, the subjects of this study were ICU nurses, as a centralized place for the admission of critically ill patients, ICU requires nurses to provide round-the-clock, uninterrupted care. However, prolonged exposure to high-intensity, high-stress work environments predisposes nurses to physical health problems such as chronic fatigue, chronic pain, gastrointestinal dysfunction, and sleep disorders, which affect work engagement and efficiency (8). Secondly, compared with other clinical departments, ICU work exposes nurses to critically ill patients for long periods, and facing high patient resuscitation and mortality rates tends to deplete the psychological resources of ICU nurses, increasing negative emotions such as psychological distress, burnout, and vicarious trauma, which leads to the inability of ICU nurses to dedicate their energy to their work thoroughly. Several previous studies have also shown that mental health status is significantly associated with presenteeism, and negative psychological emotions are risk factors for presenteeism (13, 36). In addition, most ICU in China have a group work model with clear responsibilities and a strong division of labor among team members. Therefore, when nurses suffer from poor health, most ICU nurses will continue working out of professional ethics, thus increasing the risk of presenteeism (37). Moreover, if hospitals have shortages in ICU human resource allocation, it will lead to the need for existing staff to take on more work tasks, which will not only increase their burden, but may also lead to illness or leave due to overwork. Finally, ICU nurses have frequent night shifts and high work intensity, especially female nurses who have important roles in their families, and it is difficult to coordinate work and family responsibilities, leading to significant work–family conflicts, which in turn affects physical and mental health and increases the rate of presenteeism (15). Thus, nursing managers should pay great attention to and promptly identify ICU nurses at high risk of presenteeism and focus on how to effectively respond to the influencing factors arising from presenteeism to reduce presenteeism and related adverse outcomes to improve the quality of nursing services and to protect patient safety.

### 4.2 Factors influencing presenteeism among ICU nurses

#### 4.2.1 Income per month

The results of this study showed that income per month had a significant effect on the occurrence of presenteeism, i.e., the higher the income of ICU nurses, the lower the incidence of presenteeism, which is consistent with the results of the study by Li et al. (38). In other words, presenteeism decreases as average monthly income increases, with the lowest rate among ICU nurses whose average monthly income is more significant than 11,001 yuan (approximately 1,517 U.S. dollars). As a fundamental guarantee for human survival, economic income is closely related to life satisfaction and family happiness (39). The level of average monthly income responds to the degree of matching between labor payment and reward. When labor payment is lower than financial reward, it will impair nurses’ positive psychological coping resources, reduce work enthusiasm and motivation, and lead to a decrease in the quality and efficiency of work, increasing the risk of presenteeism. In addition, when the financial income can meet the individual’s survival and development needs, the individual has more time and energy to focus on their health and sense of meaning at work and also has access to more family and social resources to support them, which improves work commitment and reduces presenteeism. Baek et al. also showed that financial income was significantly associated with presenteeism, and it was a protective factor for presenteeism (40). Another study also suggests that financial burden is an essential reason nurses choose to work despite their impaired health status (41). A survey by Yang et al. showed that nurses’ salary income in Chinese tertiary public hospitals gradually increased (42). However, there is still a gap between the actual and expected salaries, and there is a large gap between the average annual salary of nurses in developed countries, such as the United States. Therefore, hospital administrators should pay attention to the distribution system of nurses’ wages and income, establish a reasonable salary growth mechanism, and make the economic income reflect the value of nurses’ labor to improve nurses’ job satisfaction and happiness and reduce presenteeism.

#### 4.2.2 Physical health condition

Health is the first productive force, and physical health is the prerequisite and important guarantee of health productivity. Due to the unique nature of work, such as high load, high pressure, night shift rotation, irregular diet, etc., nurses are a high-risk group for impaired health productivity, which makes them often suffer from chronic fatigue, sleep disorders, chronic pain, gastrointestinal symptoms and other symptoms (43). Some studies have shown that 90% of nurses in China have sick work behaviors, resulting in an indirect economic loss of approximately 2.88 billion to 4.38 billion yuan annually (16). This study showed that the physical health condition of ICU nurses has an essential effect on presenteeism, and the worse the health status, the more serious the presenteeism, which is consistent with the results of our previous study (21). Another study showed a positive correlation between the physical health status of healthcare workers and their ability to work (44). When physical health is poor, individuals will not be able to concentrate on their work and will be prone to poor concentration, drowsiness, and fatigue, affecting work performance. In addition, physical health condition also has a particular impact on mental health; poor physical health is prone to make nurses have negative emotions such as anxiety, frustration, depression, sadness and psychological pain (45). Therefore, hospitals should establish a perfect health management system and conduct regular physical examinations and assessments for ICU nurses to promptly detect and intervene in health problems. In addition, attention should also be paid to the mental health and work pressure of ICU nurses, providing necessary psychological support and counseling, as well as reasonable work arrangements and rest time, starting from various aspects, to jointly reduce the incidence of presenteeism and guarantee the quality of ICU nursing.

#### 4.2.3 Job satisfaction

Job satisfaction is the degree of subjective satisfaction of employees with the work itself and working conditions. Some studies have shown that the job satisfaction rate of medical staff in tertiary public hospitals in China is only 40% (46). Previous studies have shown that low levels of job satisfaction are closely related to high burnout, psychological distress, and willingness to leave the job, which leads to low work engagement among nurses (47). In this study, ICU nurses were found to have a moderate level of job satisfaction (69.6% satisfaction rate.) Logistic regression analysis showed that higher job satisfaction was negatively associated with presenteeism, consistent with Rodríguez-Cifuentes et al. (48). Another systematic review also showed that job satisfaction was significantly associated with nurses’ presenteeism and willingness to leave (49). As a research area of positive psychology, job satisfaction is an essential indicator of nurses’ work-related quality of life. It responds to the extent to which nurses recognize their jobs in terms of, for example, job content, work environment, interpersonal relationships, and compensation and benefits. Relative to nurses with low job satisfaction, higher job satisfaction can stimulate nurses’ enthusiasm for work, which is conducive to enhancing work efficiency, motivation, and creativity. In addition, Penconek et al.’s systematic evaluation showed that job characteristics (e.g., interpersonal relationships, role overload, etc.), organizational characteristics (e.g., organizational culture, organizational support, etc.), and personal characteristics (e.g., personality, health status) were significantly related to nurses’ job satisfaction (50). Therefore, first, nurse administrators should create a supportive work environment, improve management and communication mechanisms, and enhance ICU nurses’ sense of involvement and belonging. Second, flexible work arrangements and adequate rest time should be provided to help ICU nurses balance work and personal life and reduce burnout. In addition, team building and staff mentoring are strengthened, and team-building activities are organized regularly to enhance mutual support and understanding among colleagues. Providing ICU nurses with continuous professional development opportunities, such as professional training and academic exchanges, increases nurses’ motivation and loyalty, which effectively reduces presenteeism and improves the quality of care and patient satisfaction.

#### 4.2.4 Perceived work stress

In clinical practice, job stress is a series of physiological, psychological, and behavioral responses caused by nurses’ job demands exceeding job resources. The cognitive interaction theory of stress suggests that whether or not a stressor actually affects an individual depends on the individual’s cognitive response and coping ability (51). Appropriate stress stimulates an individual’s intrinsic motivation to become more focused and productive in their work, facilitating the achievement of target behaviors and outcomes. When stress exceeds the range of an individual’s coping ability, it causes negative emotions such as anxiety, depression, and tension, which make them feel exhausted and unable to concentrate on their work, or even burnout and willingness to leave the job, thus affecting the quality of nurses’ professional life and the quality of nursing services, and the phenomenon of presenteeism. This study found that ICU nurses’ perceived stress was significantly correlated with presenteeism, with 63.7% of ICU nurses under high levels of work stress. Job demand-resource theory also emphasizes that when job demands (stressors) are persistently high and not compensated by job resources (coping resources) (52). Health depletion occurs, affecting work behaviors and outcomes. Compared with ordinary clinical departments, the ICU, as a platform department for centralized treatment of patients with acute and critical illnesses, requires nurses to have keen observation ability and rapid decision-making ability due to the heavy condition of patients, fast changes, and complex treatment measures. The fast-paced work style and unstable condition of patients increase nurses’ work pressure. In addition to the complexity of ICU therapeutic instruments and equipment, long-term exposure to a large number of instrument alarms is also prone to cause nurses anxiety, tension and other negative emotions, indirectly affecting the efficiency and quality of nurses’ work. At the same time, most of China’s ICU human resources (56.8%) have yet to reach the allocation of 1:2.5–3, and the shortage of human resources further increases the nurses’ workload (21). When ICU nurses are exposed to a high work-stress environment for an extended period, presenteeism, such as decreased work efficiency and quality, will occur. Therefore, managers should strengthen psychological counseling and tutoring services to help nurses master the correct methods of stress release and reasonably regulate work stress. In addition, communication and collaboration within the organization should be strengthened. An excellent interpersonal atmosphere and a humane scheduling system should be established to increase nurses’ empowering behaviors and avoid excessive overtime work and overloading of nurses so that they can maintain sufficient energy and good mental health to devote themselves to their work, improve work efficiency and quality, and reduce the occurrence of presenteeism.

#### 4.2.5 Perceived social support

Perceived social support is the emotional experience of being respected, understood, and supported by the outside world, which the individual subjectively feels. It will likely positively affect the individual’s psychological health more than objective social support (34). Previous studies have shown that low levels of perceived social support not only affect nurses’ job satisfaction and psychological wellbeing but also lead to decreased levels of organizational commitment, which affects job performance and thus increases presenteeism and willingness to leave (53). Presenteeism is a hostile work state that occurs when an individual’s work resources cannot meet the job requirements under insufficient social support. This study found that the higher the level of perceived social support, the lower the incidence of presenteeism among ICU nurses. This is consistent with the findings of Yang et al. (24). The level of social support in ICUs is critical to nurses’ physical and mental health and engagement due to the unique work’s work environment and nature. When nurses perceive a higher level of social support, they can feel care and support from the organization, colleagues and friends. They can effectively alleviate anxiety, depression and other psychological disturbances, as well as obtain emotional comfort and support so that they are full of enthusiasm and self-confidence in their work, which is conducive to reducing the incidence of presenteeism. In addition, a sound social support system makes it easier for nurses to establish a relationship of trust and respect among themselves. It enhances their sense of work identity and belonging, thus helping them to cope better with work stressors and improve work efficiency and quality. Therefore, nursing managers should create an excellent organizational and cultural atmosphere for nurses in the workplace, provide nurses with supportive and equal communication and exchange platforms, and build an all-round and multi-level social support system of “hospital-society-family” to protect nurses’ physical and mental health and continuously improve their professionalism. Physical and psychological health constantly improves nurses’ professional development ability and professionalism, enhances the sense of professional belonging and value, and reduces presenteeism.

#### 4.2.6 Transformational leadership

This study found that presenteeism of ICU nurses was negatively associated with transformational leadership, and the Labrague et al. study came up with similar results (23). Transformational leadership, as one of the approaches in leadership, focuses on motivating employees to accomplish work-level self-transcendence by enlightening them about the importance of task-taking, establishing an atmosphere of trust in the team, and stimulating their high-level needs. Transformational leadership emphasizes the leader’s charisma, convening power, intellectual stimulation, and personalized care (54). Previous studies have shown that transformational leadership is closely related to nurses’ work wellbeing, innovative behaviors, and psychological resilience, reduces burnout, and improves patient clinical safety outcomes (55). A systematic evaluation by Ystaas et al. showed that transformational leadership reduces the culture of blame in the nursing work environment and promotes a culture of safety (56). Another systematic review also showed that transformational leadership behaviors, directly and indirectly, link patient care quality and safety (57). With the development of modern nursing management, nursing management in China is changing from a paternalistic leadership style to a transformational one, from emphasizing discipline and norms to focusing on nurses’ proactive behaviors in managerial behavior. Transformational leadership style, as a positive and forward-looking leadership style, focuses on stimulating nurses’ high-level needs, which is more capable of mobilizing nurses’ motivation and enthusiasm and enhancing nurses’ sense of belonging and teamwork, thus increasing work motivation and creativity. In fact, the higher the transformational leadership, the more ICU nurses can feel the leader’s humanized care and charisma, which is conducive to improving work efficiency and service quality. In addition, transformational leadership is closely related to the willingness to leave, which impacts work engagement (58). Therefore, hospital administrators should be aware of the importance of leadership style on ICU nurses’ work behaviors and outcomes and establish a transformational leadership team to enhance nurses’ motivation and creativity and reduce presenteeism and related adverse outcomes. In addition, a global leadership training program can improve nurses’ clinical leadership and enhance their proactive work behaviors.

#### 4.2.7 Occupational coping self-efficacy

In this study, high levels of occupational coping self-efficacy were also negatively associated with presenteeism, consistent with our previous findings (21). Occupational coping self-efficacy as nurses’ confidence in their ability to cope with the demands of nursing work is a self-subjective perception and evaluation. Laschinger et al. showed that improving occupational coping self-efficacy can help nurses cope with their work needs and reduce burnout (59). Another study showed that occupational coping self-efficacy is protective of willingness to leave a job (60). In addition, studies have shown that occupational coping self-efficacy also relieves nurses’ psychological distress and improves work wellbeing, resilience, and post-traumatic growth (61). ICU nurses face complex disease treatment tasks and undertake patients’ daily primary nursing care and caregiving work; the tedious tasks are prone to reducing nurses’ sense of professional achievement and identity, and burnout occurs. When ICU nurses have a sense of confusion and helplessness in clinical work full of uncertainty and complexity and are not confident in their self-worth and stress-coping ability, they tend to choose coping methods such as withdrawal and avoidance, resulting in a lack of work attention and energy. On the contrary, ICU nurses with high occupational coping self-efficacy are confident in establishing good interpersonal relationships with colleagues and dealing with work stress, tend to perform their work duties with positive beliefs, and can positively transform stress in the face of stressors by actively seeking support from the organization, colleagues, and families, thus adopting positive coping styles and are more likely to cope with challenges at work successfully (62). Therefore, first, hospitals should provide systematic training programs that not only cover clinical skills, but also include the development of skills such as stress management and emotional regulation to increase the confidence and coping ability of ICU nurses when facing challenges. Second, regular practice sharing sessions should be organized to encourage ICU nurses to share successful cases and coping strategies, and to collectively improve their problem-solving skills. In addition, management should provide timely positive feedback and recognition, emphasize the importance of personal growth and team contribution, motivate nurses to face professional challenges positively, and improve their sense of professional accomplishment and self-efficacy to effectively reduce the incidence of presenteeism.

### 4.3 Construction and application of a predictive model for presenteeism risk of ICU nurses

This study is the first to develop a risk prediction model for assessing presenteeism among ICU nurses. In this study, a predictive model for ICU nurses’ presenteeism was created from logistic regression analysis results, including seven predictors: average monthly personal income, physical health condition, job satisfaction, perceived work stress, perceived social support, transformational leadership, and occupational coping self-efficacy. Each predictor was visualized by drawing a column-line graph to more intuitively reflect the probability of presenteeism among ICU nurses. In addition, the area under the ROC curve of the model constructed in this study was 0.821, with high sensitivity and specificity and good predictive efficacy. The Hosmer-Le-meshow test showed *χ*^2^ = 5.134, *p* = 0.743, and the calibration curve plot showed that the observed curve fitted the predicted curve well, which indicated that the model had good consistency. Internal validation showed that the area under the ROC curve of the model was 0.786. The differentiation and sensitivity were good, and the model-predicted results agreed with the observations. The clinical decision curve also shows that the model has good clinical utility, which can provide a reference basis for the early identification of high presenteeism and for developing targeted intervention programs.

## 5 Limitations

This study constructed a predictive model for the risk of presenteeism among ICU nurses, but some limitations exist. First, the occurrence mechanism and influencing factors of presenteeism are complex. This study only included factors that may have a high incidence of presenteeism among ICU nurses from the perspective of literature analysis, which may have incomplete inclusion of influencing factors and needs to be further analyzed by systematically and comprehensively incorporating more influencing factors to improve the accuracy of the prediction model further. Second, this study only carried out the internal validation of the model and only selected ICU nurses in Sichuan Province, China, as the study population, which may lack a certain degree of representativeness in terms of samples and external validation with multi-center and large samples is needed in the future to evaluate better and promote the model. In addition, the cross-sectional survey used in this study and the data information was collected through the Internet, so the data results may have a particular bias. In the future, the data can be collected face-to-face to improve its accuracy.

## 6 Conclusion

The results of this study showed that income per month, physical health condition, job satisfaction, perceived work stress, perceived social support, transformational leadership, and occupational coping self-efficacy were independent risk factors for presenteeism among ICU nurses. The risk prediction model about ICU nurses’ presenteeism constructed in this study has good differentiation, calibration, and clinical validity, which is conducive to the early identification of high presenteeism risk groups by nursing managers and helps nursing managers to effectively carry out human resource management, improve nurses’ work efficiency and quality, and reduce ICU nurses’ presenteeism.

## Data Availability

The raw data supporting the conclusions of this article will be made available by the authors without undue reservation.

## References

[1] Youn H Lee M Jang SJ. Person-centred care among intensive care unit nurses: a cross-sectional study. Intensive Crit Care Nurs. (2022) 73:103293. doi: 10.1016/j.iccn.2022.103293, PMID: 35871960

[2] Quesada-Puga C Izquierdo-Espin FJ Membrive-Jiménez MJ Aguayo-Estremera R Cañadas-De La Fuente GA Romero-Béjar JL. Job satisfaction and burnout syndrome among intensive-care unit nurses: a systematic review and meta-analysis. Intensive Crit Care Nurs. (2024) 82:103660. doi: 10.1016/j.iccn.2024.103660, PMID: 38394983

[3] Sang S Wang J Jin J. Prevalence of low back pain among intensive care nurses: a meta-analysis. Nurs Crit Care. (2021) 26:476–84. doi: 10.1111/nicc.12646, PMID: 34036704

[4] Xie W Chen L Feng F Okoli CTC Tang P Zeng L. The prevalence of compassion satisfaction and compassion fatigue among nurses: a systematic review and meta-analysis. Int J Nurs Stud. (2021) 120:103973. doi: 10.1016/j.ijnurstu.2021.103973, PMID: 34102372

[5] De Swardt C Fouché N. “What happens behind the curtains?” An exploration of ICU nurses' experiences of post mortem care on patients who have died in intensive care. Intensive Crit Care Nurs. (2017) 43:108–15. doi: 10.1016/j.iccn.2017.05.005, PMID: 28595826

[6] Min A Kang M Park H. Global prevalence of presenteeism in the nursing workforce: a meta-analysis of 28 studies from 14 countries. J Nurs Manag. (2022) 30:2811–24. doi: 10.1111/jonm.13688, PMID: 35593655

[7] Liu X Jia P Wen X Huang X Wu J. Analysis of the current situation and influencing factors of presenteeism of ICU nurses in China. J Nurs. (2022) 29:1–5. doi: 10.16460/j.issn1008-9969.2022.16.001

[8] Rainbow JG Drake DA Steege LM. Nurse health, work environment, presenteeism and patient safety. West J Nurs Res. (2020) 42:332–9. doi: 10.1177/0193945919863409, PMID: 31296124

[9] Chapman LS. Presenteeism and its role in worksite health promotion. Am J Health Promot. (2005) 19:1–15. doi: 10.4278/0890-1171-19.4.TAHP-1, PMID: 15768928

[10] Guo S Zhang H Chang Y Zhang J Chen H Zhang L. The relationship between presenteeism among nurses and patients' experience in tertiary hospitals in China. Heliyon. (2023) 9:e22097. doi: 10.1016/j.heliyon.2023.e22097, PMID: 38107301 PMC10724535

[11] Mohammadi MM Nayeri ND Varaei S Rasti A. The nurse without a nurse: the antecedents of presenteeism in nursing. BMC Nurs. (2021) 20:143. doi: 10.1186/s12912-021-00669-1, PMID: 34389006 PMC8361635

[12] Pereira F Querido AI Bieri M Verloo H Laranjeira CA. Presenteeism among nurses in Switzerland and Portugal and its impact on patient safety and quality of care: protocol for a qualitative study. JMIR Res Protoc. (2021) 10:e27963. doi: 10.2196/27963, PMID: 33983134 PMC8160804

[13] Fiorini LA Houdmont J Griffiths A. Nurses' perceived work performance and health during presenteeism: cross-sectional associations with personal and organisational factors. J Nurs Manag. (2022) 30:O37–o45. doi: 10.1111/jonm.13065, PMID: 32506664

[14] Rantanen I Tuominen R. Relative magnitude of presenteeism and absenteeism and work-related factors affecting them among health care professionals. Int Arch Occup Environ Health. (2011) 84:225–30. doi: 10.1007/s00420-010-0604-5, PMID: 21140162

[15] Ren Z Sun Y Li X He M Shi H Zhao H. How do presenteeism and family functioning affect the association between Chinese Nurses' job stress and intention to stay? J Am Psychiatr Nurses Assoc. (2024) 30:559–68. doi: 10.1177/10783903221140329, PMID: 36457173

[16] Shan G Wang S Wang W Guo S Li Y. Presenteeism in nurses: prevalence, consequences, and causes from the perspectives of nurses and chief nurses. Front Psych. (2020) 11:584040. doi: 10.3389/fpsyt.2020.584040 PMC7819974 33488418

[17] Koopman C Pelletier KR Murray JF Sharda CE Berger ML Turpin RS. Stanford presenteeism scale: health status and employee productivity. J Occup Environ Med. (2002) 44:14–20. doi: 10.1097/00043764-200201000-00004, PMID: 11802460

[18] Lerner D Amick BC 3rd Rogers WH Malspeis S Bungay K Cynn D. The work limitations questionnaire. Med Care. (2001) 39:72–85. doi: 10.1097/00005650-200101000-00009, PMID: 11176545

[19] Durmuş A Ünal Ö Türktemiz H Öztürk YE. The effect of nurses' perceived workplace incivility on their presenteeism and turnover intention: the mediating role of work stress and psychological resilience. Int Nurs Rev. (2024) 71:960–8. doi: 10.1111/inr.12950, PMID: 38465769 PMC11600495

[20] Fan S Zhou S Ma J An W Wang H Xiao T. The role of the nursing work environment, head nurse leadership and presenteeism in job embeddedness among new nurses: a cross-sectional multicentre study. BMC Nurs. (2024) 23:159. doi: 10.1186/s12912-024-01823-1, PMID: 38443951 PMC10913553

[21] Wu J Li Y Lin Q Zhang J Liu Z Liu X. The effect of occupational coping self-efficacy on presenteeism among ICU nurses in Chinese public hospitals: a cross-sectional study. Front Psychol. (2024) 15:1347249. doi: 10.3389/fpsyg.2024.1347249, PMID: 38356774 PMC10865889

[22] Yang T Ma M Guo Y Li Y Tian H Liu Y. Do job stress, health, and presenteeism differ between Chinese healthcare workers in public and private hospitals: a cross sectional study. Psychol Health Med. (2020) 25:653–65. doi: 10.1080/13548506.2019.1668564 31537117

[23] Labrague LJ Nwafor CE Tsaras K. Influence of toxic and transformational leadership practices on nurses' job satisfaction, job stress, absenteeism and turnover intention: a cross-sectional study. J Nurs Manag. (2020) 28:1104–13. doi: 10.1111/jonm.13053, PMID: 32453901

[24] Yang T Ma T Liu P Liu Y Chen Q Guo Y. Perceived social support and presenteeism among healthcare workers in China: the mediating role of organizational commitment. Environ Health Prev Med. (2019) 24:55. doi: 10.1186/s12199-019-0814-8, PMID: 31481032 PMC6724257

[25] Kang X Yang L Xu L Yue Y Ding M. Latent classes of circadian type and presenteeism and work-related flow differences among clinical nurses: a cross-sectional study. Psychiatry Investig. (2022) 19:311–9. doi: 10.30773/pi.2021.0357, PMID: 35500904 PMC9058268

[26] Jianlan R Mei Y Chunyan Y Rendie X Yiping B Li L. Exploring anesthesiology nurse' presenteeism in China: cross-sectional study. BMC Public Health. (2024) 24:2008. doi: 10.1186/s12889-024-19476-9, PMID: 39060992 PMC11282700

[27] Xie W Liu M Okoli CTC Zeng L Huang S Ye X. Construction and evaluation of a predictive model for compassion fatigue among emergency department nurses: a cross-sectional study. Int J Nurs Stud. (2023) 148:104613. doi: 10.1016/j.ijnurstu.2023.104613, PMID: 37839306

[28] Zhang X Zhang L. Risk prediction of sleep disturbance in clinical nurses: a nomogram and artificial neural network model. BMC Nurs. (2023) 22:289. doi: 10.1186/s12912-023-01462-y, PMID: 37641040 PMC10463587

[29] Mo YH Su YD Dong X Zhong J Yang C Deng WY. Development and validation of a nomogram for predicting sarcopenia in community-dwelling older adults. J Am Med Dir Assoc. (2022) 23:715–721.e5. doi: 10.1016/j.jamda.2021.11.023, PMID: 34932988

[30] Bandura A. Social cognitive theory: an agentic perspective. Annu Rev Psychol. (2001) 52:1–26. doi: 10.1146/annurev.psych.52.1.1, PMID: 11148297

[31] Peduzzi P Concato J Feinstein AR Holford TR. Importance of events per independent variable in proportional hazards regression analysis. II. Accuracy and precision of regression estimates. J Clin Epidemiol. (1995) 48:1503–10. doi: 10.1016/0895-4356(95)00048-8, PMID: 8543964

[32] Li C Shi K. The structure and measurement of transformational leadership in China. Acta Psychol Sin. (2005) 6:97–105.

[33] Pisanti R Lombardo C Lucidi F Lazzari D Bertini M. Development and validation of a brief occupational coping self-efficacy questionnaire for nurses. J Adv Nurs. (2008) 62:238–47. doi: 10.1111/j.1365-2648.2007.04582.x, PMID: 18394036

[34] Blumenthal JA Burg MM Barefoot J Williams RB Haney T Zimet G. Social support, type a behavior, and coronary artery disease. Psychosom Med. (1987) 49:331–40. doi: 10.1097/00006842-198707000-00002, PMID: 3615762

[35] Freeling M Rainbow JG Chamberlain D. Painting a picture of nurse presenteeism: a multi-country integrative review. Int J Nurs Stud. (2020) 109:103659. doi: 10.1016/j.ijnurstu.2020.103659, PMID: 32585449

[36] Gustafsson K Marklund S Leineweber C Bergström G Aboagye E Helgesson M. Presenteeism, psychosocial working conditions and work ability among care workers-a cross-sectional Swedish population-based study. Int J Environ Res Public Health. (2020) 17:2419. doi: 10.3390/ijerph17072419, PMID: 32252368 PMC7177781

[37] Chiu S Black CL Yue X Greby SM Laney AS Campbell AP. Working with influenza-like illness: presenteeism among US health care personnel during the 2014-2015 influenza season. Am J Infect Control. (2017) 45:1254–8. doi: 10.1016/j.ajic.2017.04.008, PMID: 28526310 PMC5670002

[38] Li Y Guo B Wang Y Lv X Li R Guan X. Serial-multiple mediation of job burnout and fatigue in the relationship between sickness presenteeism and productivity loss in nurses: a multicenter cross-sectional study. Front Public Health. (2021) 9:812737. doi: 10.3389/fpubh.2021.812737 35096756 PMC8795673

[39] Albashayreh A Al Sabei SD Al-Rawajfah OM Al-Awaisi H. Healthy work environments are critical for nurse job satisfaction: implications for Oman. Int Nurs Rev. (2019) 66:389–95. doi: 10.1111/inr.12529, PMID: 31206654

[40] Baek J Ki J Ryu J Smi CK. Relationship between occupational stress, sleep disturbance, and presenteeism of shiftwork nurses. J Nurs Scholarsh. (2022) 54:631–8. doi: 10.1111/jnu.12766, PMID: 35084088

[41] Laranjeira C Pereira F Querido A Bieri M Verloo H. Contributing factors of presenteeism among Portuguese and Swiss nurses: a qualitative study using focus groups. Int J Environ Res Public Health. (2022) 19:8844. doi: 10.3390/ijerph19148844, PMID: 35886694 PMC9316472

[42] Yang S Zhang X Wu Y Liu M Sun J Hu L. Analysis of the current situation and trend of nurses' salary level and satisfaction rate in tertiary public hospitals in China. Chinese J Hosp Manag. (2021) 37:488–93. doi: 10.3760/cma.j.cn111325-20210506-00389

[43] Nwosu ADG Ossai E Onwuasoigwe O Ezeigweneme M Okpamen J. Burnout and presenteeism among healthcare workers in Nigeria: implications for patient care, occupational health and workforce productivity. J Public Health Res. (2021) 10:1900. doi: 10.4081/jphr.2021.1900 33634041 PMC7883015

[44] Garzaro G Clari M Ciocan C Albanesi B Guidetti G Dimonte V. Physical health and work ability among healthcare workers. A cross-sectional study. Nurs Rep. (2022) 12:259–69. doi: 10.3390/nursrep12020026, PMID: 35466246 PMC9036298

[45] Aboagye E Björklund C Gustafsson K Hagberg J Aronsson G Marklund S. Exhaustion and impaired work performance in the workplace: associations with presenteeism and absenteeism. J Occup Environ Med. (2019) 61:e438–44. doi: 10.1097/JOM.0000000000001701, PMID: 31478995

[46] Yang S Ma J Wu Y Chen X Pei C Cao M. A survey to assess the overall satisfaction rate of medical and nursing staff with their jobs in tertiary public hospitals in China. China Res Hosp. (2020) 7:42–46+155–160.

[47] Waltz LA Muñoz L Weber Johnson H Rodriguez T. Exploring job satisfaction and workplace engagement in millennial nurses. J Nurs Manag. (2020) 28:673–81. doi: 10.1111/jonm.12981, PMID: 32068932

[48] Rodríguez-Cifuentes F Fernández-Salinero S Moriano JA Topa G. Presenteeism, overcommitment, workplace bullying, and job satisfaction: a moderated mediation relationship. Int J Environ Res Public Health. (2020) 17:8616. doi: 10.3390/ijerph17228616, PMID: 33233538 PMC7699487

[49] Lu H Zhao Y While A. Job satisfaction among hospital nurses: a literature review. Int J Nurs Stud. (2019) 94:21–31. doi: 10.1016/j.ijnurstu.2019.01.011, PMID: 30928718

[50] Penconek T Tate K Bernardes A Lee S Micaroni SPM Balsanelli AP. Determinants of nurse manager job satisfaction: a systematic review. Int J Nurs Stud. (2021) 118:103906. doi: 10.1016/j.ijnurstu.2021.103906, PMID: 33765624

[51] Lee SA Mathis AA Jobe MC Pappalardo EA. Clinically significant fear and anxiety of COVID-19: a psychometric examination of the coronavirus anxiety scale. Psychiatry Res. (2020) 290:113112. doi: 10.1016/j.psychres.2020.113112, PMID: 32460185 PMC7237368

[52] Bakker AB Demerouti E. Job demands-resources theory: taking stock and looking forward. J Occup Health Psychol. (2017) 22:273–85. doi: 10.1037/ocp0000056, PMID: 27732008

[53] Coffeng JK Hendriksen IJ Duijts SF Twisk JW Van Mechelen W Boot CR. Effectiveness of a combined social and physical environmental intervention on presenteeism, absenteeism, work performance, and work engagement in office employees. J Occup Environ Med. (2014) 56:258–65. doi: 10.1097/JOM.0000000000000116, PMID: 24603201

[54] Lui JNM Andres EB Johnston JM. How do organizational culture and leadership style affect nurse presenteeism and productivity?: a cross sectional study of Hong Kong acute public hospitals. Int J Nurs Stud. (2024) 152:104675. doi: 10.1016/j.ijnurstu.2023.104675 38277926

[55] Abdul Salam H Dumit NY Clinton M Mahfoud Z. Transformational leadership and predictors of resilience among registered nurses: a cross-sectional survey in an underserved area. BMC Nurs. (2023) 22:37. doi: 10.1186/s12912-023-01192-1, PMID: 36759906 PMC9912636

[56] Ystaas LMK Nikitara M Ghobrial S Latzourakis E Polychronis G Constantinou CS. The impact of transformational leadership in the nursing work environment and patients' outcomes: a systematic review. Nurs Rep. (2023) 13:1271–90. doi: 10.3390/nursrep13030108, PMID: 37755351 PMC10537672

[57] Alanazi NH Alshamlani Y Baker OG. The association between nurse managers' transformational leadership and quality of patient care: a systematic review. Int Nurs Rev. (2023) 70:175–84. doi: 10.1111/inr.12819, PMID: 36583960

[58] Wu X Hayter M Lee AJ Yuan Y Li S Bi Y. Positive spiritual climate supports transformational leadership as means to reduce nursing burnout and intent to leave. J Nurs Manag. (2020) 28:804–13. doi: 10.1111/jonm.12994, PMID: 32145113

[59] Laschinger HK Borgogni L Consiglio C Read E. The effects of authentic leadership, six areas of worklife, and occupational coping self-efficacy on new graduate nurses' burnout and mental health: a cross-sectional study. Int J Nurs Stud. (2015) 52:1080–9. doi: 10.1016/j.ijnurstu.2015.03.002, PMID: 25801311

[60] Fallatah F Laschinger HK Read EA. The effects of authentic leadership, organizational identification, and occupational coping self-efficacy on new graduate nurses' job turnover intentions in Canada. Nurs Outlook. (2017) 65:172–83. doi: 10.1016/j.outlook.2016.11.020, PMID: 28126250

[61] Pisanti R Van Der Doef M Maes S Lombardo C Lazzari D Violani C. Occupational coping self-efficacy explains distress and well-being in nurses beyond psychosocial job characteristics. Front Psychol. (2015) 6:1143. 26300827 10.3389/fpsyg.2015.01143 PMC4526791

[62] Lin Q Fu M Sun K Liu L Chen P Li L. The mediating role of perceived social support on the relationship between lack of occupational coping self-efficacy and implicit absenteeism among intensive care unit nurses: a multicenter cross-sectional study. BMC Health Serv Res. (2024) 24:653. doi: 10.1186/s12913-024-11084-y, PMID: 38773420 PMC11110179

[63] Warren CL White-Means SI Wicks MN Chang CF Gourley D Rice M. Cost burden of the presenteeism health outcome: diverse workforce of nurses and pharmacists. J Occup Environ Med. (2011): 90–9. doi: 10.1097/JOM0b013e3182028d38, PMID: 21187792

